# Using accelerometers and global positioning system devices to assess gender and age differences in children’s school, transport, leisure and home based physical activity

**DOI:** 10.1186/1479-5868-11-8

**Published:** 2014-01-24

**Authors:** Charlotte D Klinker, Jasper Schipperijn, Hayley Christian, Jacqueline Kerr, Annette K Ersbøll, Jens Troelsen

**Affiliations:** 1 Department of Sports Science and Clinical Biomechanics, University of Southern Denmark, Campusvej 55, 5230 Odense M, Denmark; 2 Centre for the Built Environment and Health, School of Population Health, and Telethon Institute for Child Health Research, The University of Western Australia, 35 Stirling Highway, Crawley, Western Australia 6009, Australia; 3 Department of Family and Preventive Medicine, University of California San Diego, La Jolla, California, USA; 4 National Institute of Public Health, University of Southern Denmark, Øster Farimagsgade 5A, 1353 Copenhagen K, Denmark

**Keywords:** Children, Adolescent, Physical activity, Accelerometer, Global positioning system (GPS), Spatial behaviour, Context-specific, Physical activity patterns

## Abstract

**Background:**

Knowledge on domain-specific physical activity (PA) has the potential to advance public health interventions and inform new policies promoting children’s PA. The purpose of this study is to identify and assess domains (leisure, school, transport, home) and subdomains (e.g., recess, playgrounds, and urban green space) for week day moderate to vigorous PA (MVPA) using objective measures and investigate gender and age differences.

**Methods:**

Participants included 367 Danish children and adolescents (11–16 years, 52% girls) with combined accelerometer and Global Positioning System (GPS) data (mean 2.5 days, 12.7 hrs/day). The Personal Activity and Location Measurement System and a purpose-built database assessed data in 15-second epochs to determine PA and assign epochs to 4 domains and 11 subdomains. Frequencies and proportions of time spent in MVPA were determined and differences assessed using multi-level modeling.

**Results:**

More than 90% of MVPA was objectively assigned to domains/subdomains. Boys accumulated more MVPA overall, in leisure, school and transport (all *p* < 0.05). Children compared with adolescents accumulated more MVPA, primarily through more school MVPA (*p* < 0.05). Boys spent a large proportion of time accumulating MVPA in playgrounds, active transport, Physical Education, sports facilities, urban green space and school grounds. Girls spent a significant proportion of time accumulating MVPA in active transport and playgrounds. No gender or age differences were found in the home domain.

**Conclusions:**

Large variations were found in PA frequency and intensity across domains/subdomains. Significant gender differences were found, with girls being less active in almost all domains and subdomains. Objectively measured patterns of PA across domains/subdomains can be used to better tailor PA interventions and inform future policies for promoting child PA.

## Background

Physical activity (PA) is internationally recognised as an important component of a child and adolescent’s health – contributing to better current and future physical, mental and social health [1]. Globally, many children are not sufficiently physically active to maintain good health [2] and often PA declines during adolescence making this period particularly important for health promotion efforts [3,4]. The SLOTH model highlights four domains which are important for PA promotion among children: leisure, school, transport and home [5]. Little is known about the nature of PA patterns in domains and how this varies by child age and gender. Knowledge of domain specific PA patterns is warranted [6] to guide more targeted public health interventions and inform policies aimed at promoting child and adolescent PA.

The development of lightweight, affordable Global Positioning System (GPS) devices has made it possible to record and describe domains and locations of children’s PA [7-12]. Wearing accelerometers and GPS devices simultaneously allows objective measurement of the amount of time and PA in domains, as well as investigation of gender and age differences in “spatial behavior” (individual patterns of movement in space) [13]. A handful of studies have used objective measures to describe the spatial activity pattern among children and adolescents [10,12,14], but to date none have used a theoretical domain focused approach. Moreover, it is unclear if particular subdomains within each domain are more or less important targets for PA intervention. Subdomains can be locations, facilities or organizational constructs within domains. Recess and Physical Education (PE) are examples of organizational opportunities that can be used to promote PA during school hours [15]. Locations such as playgrounds [16,17], urban green space [9,11] or facilities such as sports facilities [18] are potential subdomains for PA during leisure time. To date it appears that no studies have investigated both the amount of moderate to vigorous PA (MVPA) and the proportion of time spent in MVPA [19] in domains and subdomains.

The purpose of this study was to use combined accelerometer and GPS measurements to: 1) Identify gender and age differences in MVPA across domains and subdomains, 2) Determine the amount of MVPA undertaken in domains (leisure, school, transport, and home) and subdomains (e.g. recess, PE, playgrounds, sports facilities and urban green space), and to 3) Determine the proportion of time spent in MVPA in domains and subdomains.

## Methods

### Study population and procedures

The When Cities Move Children (WCMC) study is a longitudinal natural experiment conducted in and around the Haraldsgade district in Copenhagen, the capital of Denmark. Approximately 9,300 people reside in the Haraldsgade district, 45% in public housing estates (compared with 19% in the municipality of Copenhagen), and one out of three are immigrants or descendants of immigrants (compared with 19% in the municipality of Copenhagen and 10% in Denmark) [20-22]. Approximately 1800 children live in the area of which 200 are immigrants and 1100 descendants of immigrants [20,21]. The WCMC study evaluates the effects of changes to the built environment on PA and movement patterns. This study uses data from the baseline data collection 2010–11. All children in grade 5–8 from four schools were invited to participate. A passive informed consent procedure was used, where students were included unless the parents withdrew consent. This procedure has been found to be ethically appropriate in low-risk research in adolescents [23]. The Danish Ethical Regional Committee reviewed the study protocol and concluded that formal ethics approval was not required. The study is registered and approved by the Danish Data Protection Agency (reference number: 2009-41-3943).

### Physical activity and spatial movement measures

Participants were asked to wear an Actigraph GT3X Activity accelerometer and a QStarz BT-Q1000X GPS device [24,25] on a belt on the hip (opposite sides) for five weekdays and two weekend days [26]. Participants were instructed to charge the GPS every night, to wear the monitors during waking hours and remove them only for bathing, or water sports. The GPS was set to record data at 15-second epochs using Qstarz software. The accelerometers were initialized using 2-second epochs using ActiLife software (Version 4.4), and later aggregated to 15-second epochs using the Personal Activity and Location Measurement System (PALMS) [27,28]. PALMS processed the accelerometer data to provide values for MVPA using 15-second cut points [29,30]. Continuous periods of at least 60 minutes of zero values were classified as non-wear time, and removed. PALMS processed the GPS data by identifying invalid data points using extreme speed or changes in distance and elevation, and replaced invalid points by imputing data from the last known valid point (up to 10 minutes). Processed GPS data were matched to accelerometer data in 15-second epochs.

### Covariates

Information on ethnicity was obtained from Statistics Denmark using a unique Danish personal identification number [31] and children were categorised as Danish or immigrant/descendent from immigrants. Parent employment status was obtained from registers on personal labour market affiliation [32] and parents were categorised as working or one (or both) parents unemployed. Grade was used as a proxy for age and dichotomized into grade 5–6 versus grade 7–8 to approximate the life stages ‘childhood’ and ‘adolescence’. An E-survey was conducted at the same time as the accelerometer and GPS data collection. The survey assessed BMI (self-report height and weight [33]) and for those children whose parents were separated, their place of residence during data collection. Participants also completed a daily diary during all seven days of data collection to assess changes to the regular school schedule.

### Participants

Across the four schools 623 children were invited to take part in the study and consent was obtained from 523 (83.9%). During data collection 14 lost one or both devices or returned malfunctioning devices reducing the sample to 509 participants of whom 422 participants fulfilled the inclusion criteria of having at least one valid day of nine hours combined accelerometer and GPS data. Data from day one were later removed as the equipment was fitted at different times during the school day. Days where children accumulated no GPS time at home were inspected manually. When the E-survey revealed that data belonged to a child with separated parents, and that the specific day was not spent at the primary parent’s home, it was removed. Finally, weekend data was removed leaving a total sample of 367 participants with valid accelerometer and GPS data (72.1%). Only week day data was included in the current study as this provided the greatest variability in domains and subdomains.

### Domains, subdomains and outcome variables

A purpose built PostgreSQL database combined the PALMS dataset with Geographic Information System (GIS) data and information on school schedules, including individual deviations, to compute the outcome variables. Minutes of MVPA (outcome 1) and proportion of time spent in MVPA (outcome 2) were determined for four domains: leisure, school, transport and home, and 11 subdomains: clubs, sports facilities, playgrounds, urban green space, shopping centres, school grounds, ‘other places’, total recess, PE, and active (walking, biking) and passive (vehicle) transport. Table 1 shows the definitions and procedures used to assess domains and subdomains. The domains and subdomains investigated in this study were based on the literature and reflected places where children and adolescents are typically involved in MVPA [5,10,12,14]. School schedules and participant diaries, depicting changes to the ordinary schedule as well as individual changes (e.g., PE cancelled, absent from or late for school) were used to distinguish when a participant was in school (scheduled school hours), compared with being on a school ground (outside scheduled school hours). A random selection of epochs in the subdomain ‘other places’ was manually inspected in GIS and consisted predominantly of epochs spent in houses or apartments (other than their own home), or hanging out on the streets.

**Table 1:** Definitions and method of assessment for domains and subdomains

	**Category**	**Method**	**Assessment**	**n**^**a**^	**Buffer (meter)**
**Leisure**	Domain	Objective database	Sum of all leisure subdomains.	367	10
School grounds	Subdomain	Objective database	Addresses of public schools were provided by the City of Copenhagen. They were geocoded and manually digitalized in GIS.	362	10
Clubs	Subdomain	Objective database	Addresses of public schools were provided by the City of Copenhagen. They were geocoded and school grounds were digitalized manually in GIS.	239	10
Sports facilities	Subdomain	Objective database	All sports facilities within the City of Copenhagen were identified from municipal websites, and included if they offered activities for 10–16 years olds. All addresses were geocoded and manually digitalized in GIS.	251	10
Playgrounds	Subdomain	Objective database	Addresses of playgrounds were provided by the City of Copenhagen. They were geocoded and playground areas digitalized in GIS.	169	10
Urban green space	Subdomain	Objective database	All urban green spaces were available from the Danish Geodata Agency.	352	10
Shopping centers	Subdomain	Objective database	Major indoor shopping centers were identified online, and manually digitalized in GIS.	100	10
Other places	Subdomain	Unclassified epochs	Epochs not categorized as school, home, transport and other leisure sub-domains were categorized as other places.	367	n/a
**School**	Domain	School schedule adjusted for self-reported changes	An epoch was assigned to school, recess or PE according to the school schedule if a) the student diary reported it was a usual day or b) no student diary data was available (17%). Otherwise diary data was used.	365	n/a
Recess	Subdomain			360	n/a
PE	Subdomain			206	n/a
**Transport**	Domain	Objective PALMS	All epochs classified by PALMS as trips, and not part of any other domain.	366	n/a
Active	Subdomain	Objective PALMS	Trips categorized as walking or biking and not identified within another domain.	364	n/a
Passive	Subdomain	Objective PALMS	Trips categorized as vehicle and not identified within any other domain.	246	n/a
**Home**	Domain	Objective database	All students’ primary addresses were geocoded. Each home was digitalized manually in GIS. A house was defined as the parcel, an apartment was defined as the building and adjacent outdoor area.	366	10

^a^Number of participants who were present in a domain/subdomain during data collection and accummulated MVPA.

GIS, Geographical Information System; n/a, not applicable; PALMS, Personal Activity and Location Measurement System; PE, Physical Education.

### Data analyses

All analyses were performed using STATA SE12 in 2013. Descriptive statistics were calculated using frequency distributions (number (n) and proportion (%)), mean and standard deviation (SD), or median and interquartile ranges (IQR) for non-normally distributed variables. Univariate analyses were performed using χ^2^-test, *t*-test or Wilcoxon rank-sum test to: (1) Test differences in baseline characteristics between responders and non-responders; and (2) Test differences in baseline characteristics between children with complete and incomplete data. Multilevel analyses were conducted for each domain and subdomain for the two outcomes of interest based on individual means across valid days. Analyses adjusted for differences in age, gender, BMI, number of valid days (1–4), and total time accumulated in the day or domain being investigated. Each model accounted for the hierarchical nature of children being nested within schools [34]. Due to the limited number of schools included in this study it was included as a fixed effect. All 32 models had a non-normal distribution of residuals and 15 models were transformed by log transformation and 17 models by square root transformation. The transformed model p-value and the untransformed model coefficient for age and gender differences are shown. Sensitivity analyses with valid days entered as the unit of analysis, including students as a random effect to account for repeated measurements within students, were conducted, and showed no substantial changes in the overall results (i.e., estimated p-values).

## Results

There were no overall differences between responders (n = 523) and non-responders (n = 100) in gender, ethnicity or parental employment status. However, a greater dropout was seen among adolescents and in two of the four schools (*p* < 0.001). There were no differences in gender, age, BMI, parental employment status and ethnicity between participants who fulfilled the inclusion criteria (n = 367) and those who were excluded (n = 156) (all *p* > 0.1). However, the drop-out was unequally distributed between schools (*p* < 0.001). Table 2 shows the socio-demographic characteristics and accelerometer and GPS wear time of participants (n = 367). Participants had a mean of 2.5 (range 1–4) valid week days and a median of 12.7 (IQR 11.6-13.5) hours of daily combined accelerometer and GPS data, with girls accumulating more overall time compared with boys (*p* = 0.009).

**Table 2:** Descriptive characteristics of study participants (n = 367) by means of frequencies, mean (SD) and median (IQR)

	**Girls**	**Boys**	**Children**	**Adolescents**	**Total**
**Population (%)**	192 (52.3)	175 (47.7)	219 (59.7)	148 (40.3)	367 (100)
**Mean age (SD)**	13.2 (1.2)	13.1 (1.1)	**12.4 (0.7)**	**14.3 (0.7)**	13.2 (1.2)
**Mean BMI (SD)**^**a**^	18.9 (3.3)	18.6 (3.0)	**18.4 (3.3)**	**19.3 (2.8)**	18.8 (3.2)
**Parental employment status**^**b**^					
**Both parents employed (%)**	98 (52.7)	100 (59.2)	117 (55.5)	81 (56.3)	198 (55.8)
**One or both unemployed (%)**	88 (47.3)	69 (40.8)	94 (44.5)	63 (43.7)	157 (44.2)
**Ethnicity**^**c**^					
**Danish background (%)**	61 (32.4)	73 (42.4)	81 (37.5)	53 (36.8)	134 (37.2)
**Immigrant or descendent (%)**	127 (67.6)	99 (57.6)	135 (62.5)	91 (63.2)	226 (62.8)
**Median daily hours of combined accelerometer and GPS data (IQR)**	**12.9 (11.9-13.6)**	**12.5 (11.4-13.4)**	12.6 (11.9-13.5)	12.7 (11.3-13.6)	12.7 (11.6-13.5)
**Mean valid days (SD)**^**d**^	2.5 (1.1)	2.5 (1.1)	2.6 (1.1)	2.4 (1.0)	2.5 (1.1)

Bold: significant difference p < 0.05.

^a^data missing from 21 participants.

^b^data missing from 12 participants.

^c^data missing from 7 participants.

^d^Minimum one valid week day of nine hours of combined accelerometer and GPS data.

BMI, Body Mass Index; GPS, Global Positioning System; IQR, Inter Quartile Range; SD, Standard Deviation.

### Places for MVPA

More than 90% of participants accumulated MVPA within school grounds, urban green space, other places, recess, PE and active transport (Table 1). Half of both girls and boys did not accumulate any MVPA in playgrounds and shopping centres, and approximately half of girls did not accumulate any MVPA in clubs and sports facilities (Table 3). Similar proportions of both children and adolescents did not accumulate any MVPA in playgrounds and shopping centres and half of children did not accumulate any MVPA in clubs and sports facilities.

**Table 3:** Daily minutes of MVPA in domains and subdomains by gender and age

	**Girls**		**Boys**		**Adjusted difference**^**a**^	
	**Minutes**		**Minutes**		**Reference = girl**	
	**Median**	**IQR**	**Median**	**IQR**	**Minutes**	**p-value**
**Total MVPA**	54.9	(41.5-73.7)	75.2	(52.8-97.3)	**19.69**	**<0.001**
**Leisure time**	11.7	(7.7-20.2)	21.8	(11.8-38.3)	**10.72**	**<0.001**
School grounds	2.2	(1.3-4.3)	2.8	(1.5-7.3)	**2.31**	**0.002**
Clubs	0.0	(0.0-0.4)	0.2	(0.0-1.3)	**2.36**	**0.003**
Sports facilities	0.0	(0.0-0.5)	0.2	(0.0-4.8)	**2.94**	**<0.001**
Playgrounds	0.0	(0.0-0.3)	0.0	(0.0-0.5)	**0.69**	**0.011**
Urban green space	1.5	(0.3-3.6)	1.9	(0.5-4.4)	**0.42**	**0.037**
Shopping center	0.0	(0.0-0.0)	0.0	(0.0-0.0)	0.07	0.55
Other places	4.1	(2.2-7.8)	5.0	(2.6-11.3)	**1.94**	**0.004**
**School**	18.8	(13.0-25.8)	24.9	(15.9-35.6)	**6.00**	**<0.001**
Recess	6.0	(3.7-8.8)	8.3	(5.9-12.3)	**2.99**	**<0.001**
PE	14.4	(8.0-23.5)	19.5	(9.8-34.8)	**5.74**	**0.002**
**Transport**	12.3	(5.7-19.6)	12.7	(7.0-19.5)	**1.74**	**0.036**
Active	9.6	(4.5-16.2)	10.3	(5.6-15.6)	**1.57**	**0.031**
Passive	0.2	(0.0-3.5)	0.3	(0.0-4.1)	0.17	0.57
**Home**	6.5	(3.0-12.8)	4.8	(2.3-10.3)	0.96	0.88
	**Children**		**Adolescent**		**Adjusted difference**^**b**^	
	**Minutes**		**Minutes**		**Reference = child**	
	**Median**	**IQR**	**Median**	**IQR**	**Minutes**	**p-value**
**Total MVPA**	67.5	(50.4-90.4)	54.0	(37.7-77.1)	**−8.33**	**0.004**
**Leisure time**	18.0	(10.0-31.9)	12.4	(7.4-24.2)	**−1.50**	**0.047**
School grounds	3.3	(1.8-7.2)	1.8	(1.0-3.1)	**−0.88**	**0.001**
Clubs	0.0	(0.0-0.9)	0.1	(0.0-0.5)	**−0.24**	**0.034**
Sports facilities	0.0	(0.0-1.3)	0.1	(0.0-1.3)	−0.88	0.69
Playgrounds	0.0	(0.0-0.5)	0.0	(0.0-0.3)	−0.02	0.48
Urban green space	1.6	(0.3-3.6)	1.8	(0.4-4.3)	0.49	0.16
Shopping center	0.0	(0.0-0.0)	0.0	(0.0-0.0)	0.04	0.23
Other places	4.7	(2.3-10.0)	4.4	(2.4-8.8)	−0.01	0.56
**School**	23.9	(17.1-34.9)	17.5	(12.1-25.5)	**−5.88**	**<0.001**
Recess	7.8	(5.3-11.7)	5.7	(3.3-8.3)	**−2.40**	**<0.001**
PE	14.0	(7.8-29.3)	18.0	(10.6-25.3)	−1.99	0.68
**Transport**	12.1	(6.0-18.4)	13.4	(6.3-21.2)	0.57	0.90
Active	9.9	(5.5-15.3)	10.1	(4.5-17.4)	0.24	0.66
Passive	0.1	(0.0-3.4)	0.6	(0.0-4.5)	0.33	0.38
**Home**	5.6	(2.6-13.3)	5.5	(2.6-10.4)	−2.82	0.05

Bold: significant difference p < 0.05.

^a^Differences were estimated using multilevel adjusted for differences in age, BMI, number of valid days & time in domain. School included to account for clustering.

^b^Differences were estimated using multilevel adjusted for differences in gender, BMI, number of valid days & time in domain. School included to account for clustering.

IQR, Inter Quartile Range; MVPA, Moderate to Vigorous Physical Activity, PE, Physical Education.

### Minutes of MVPA

Boys spent almost 20 minutes longer than girls in MVPA across the day (*p* < 0.001) (Table 3), with 10.7 more minutes in MVPA in leisure (*p* < 0.001), 6.0 more minutes in MVPA in school (*p* < 0.001) and 1.7 more minutes in MVPA in transport (*p* < 0.05) domains. During leisure, boys accumulated more minutes of MVPA than girls on e.g. school grounds (2.3 minutes, *p* < 0.05), in sports facilities (2.9 minutes, *p* < 0.001) and in other places (1.9 minutes, *p* < 0.05). In school, boys spent more time in MVPA than girls both during recess (3.0 more minutes, *p* < 0.001) and PE (5.7 more minutes, *p* < 0.05). Children accumulated 8.3 more minutes of MVPA than adolescents during the day (*p* < 0.05). The majority of this difference occurred during school hours (Table 3). There were no gender or age differences in MVPA accumulated at home.

### Proportion of time spent in MVPA

The proportion of time spent in MVPA during the day was higher in boys compared with girls (*p* < 0.001) (Table 4). Significant gender differences were also found in the proportion of time spent in MVPA in leisure, school and transport domains (all *p* < 0.001). For nine out of the 11 subdomains the proportion of time spent in MVPA was higher among boys than girls. Among boys, the largest proportion of time spent in MVPA was at playgrounds (35.4%), during active transport (34.7%), PE (25.8%), in sports facilities (24.5%), urban green space (23.8%) or at school grounds (20.7%). Among girls comparable proportions of time spent in MVPA was only seen for the subdomains active transport (31.3%) and at playgrounds (20.7%). The proportion of time spent in MVPA was higher in children compared with adolescents over a whole day (*p* < 0.05), at school (*p* < 0.001) and during recess (*p* < 0.001) (Table 4). A large proportion of children’s time was classified as MVPA when in active transport (35.1%), at playgrounds (28.2%) and in PE (21.5%). Among adolescents, a greater proportion of time spent in MVPA occurred at playgrounds (33.3%), during active transport (30.0%), in urban green space (22.2%) and PE (20.5%). No gender or age differences were found in the proportion of time spent in MVPA in the home domain.

**Table 4:** Proportion of time spent in MVPA when in domains and subdomains by age and gender

	**Girls**		**Boys**		**Adjusted difference**^**a**^	
	**%**		**%**		**Reference = girl**	
	**Median**	**IQR**	**Median**	**IQR**	**%**	**p-value**
**Total MVPA**	6.7	(4.9-8.5)	9.1	(6.5-12.2)	**2.4**	**<0.001**
**Leisure time**	5.4	(3.7-8.5)	8.7	(4.9-15.3)	**4.2**	**<0.001**
School grounds	12.5	(7.2-20.5)	20.7	(13.0-31.7)	**9.3**	**<0.001**
Clubs	9.5	(0.0-50.0)	14.8	(3.9-50.0)	7.2	0.09
Sports facilities	13.2	(0.0-38.1)	24.3	(0.0-49.4)	**8.5**	**0.018**
Playgrounds	20.7	(1.8-55.0)	35.4	(11.3-75.0)	**12.0**	**0.013**
Urban green space	17.1	(4.3-34.5)	23.8	(10.2-42.1)	**9.7**	**<0.001**
Shopping center	2.4	(0.0-14.3)	6.1	(0.0-24.8)	6.6	0.17
Other places	2.6	(1.5-4.3)	3.2	(2.0-6.4)	**1.5**	**0.008**
**School**	5.9	(4.0-8.1)	8.3	(5.6-11.4)	**2.2**	**<0.001**
Recess	10.7	(6.0-15.8)	15.8	(11.2-22.6)	**5.7**	**<0.001**
PE	16.6	(9.4-28.2)	25.8	(13.4-39.3)	**7.8**	**<0.001**
**Transport**	24.9	(14.7-40.0)	32.0	(17.2-46.6)	**6.5**	**0.001**
Active	31.3	(16.5-48.2)	34.7	(17.9-54.7)	**6.0**	**0.007**
Passive	9.6	(1.8-23.0)	15.5	(5.6-26.8)	**4.8**	**0.022**
**Home**	3.6	(1.9-5.5)	3.4	(1.6-6.9)	0.5	0.85
	**Children**		**Adolescents**		**Adjusted difference**^**b**^	
	**%**		**%**		**Reference = child**	
	**Median**	**IQR**	**Median**	**IQR**	**%**	**p-value**
**Total MVPA**	8.2	(6.2-10.8)	6.7	(4.6-9.6)	**−0.9**	**0.008**
**Leisure time**	7.1	(4.4-11.5)	5.9	(3.8-10.8)	−0.9	0.11
School grounds	18.6	(10.1-26.9)	13.5	(7.6-23.8)	−0.9	0.11
Clubs	9.6	(0.7-46.7)	19.1	(3.4-53.3)	7.5	0.08
Sports facilities	17.9	(0.0-44.6)	17.8	(0.0-45.0)	−2.1	0.94
Playgrounds	28.2	(5.6-57.1)	33.3	(10.0-75.0)	10.0	0.14
Urban green space	19.6	(6.6-35.7)	22.2	(6.5-42.7)	1.1	0.36
Shopping center	5.4	(0.0-17.9)	2.5	(0.0-15.6)	−5.1	0.60
Other places	3.1	(1.7-4.9)	2.9	(1.6-5.2)	−0.3	0.92
**School**	7.8	(5.6-10.9)	5.6	(3.9-8.0)	**−2.1**	**<0.001**
Recess	14.8	(10.6-22.2)	9.8	(5.2-15.6)	**−5.0**	**<0.001**
PE	21.5	(10.9-37.6)	20.5	(12.3-29.1)	−3.6	0.22
**Transport**	31.4	(17.7-45.2)	24.0	(12.9-39.1)	−1.9	0.27
Active	35.1	(18.8-53.9)	30.0	(14.3-48.2)	−0.3	0.61
Passive	13.3	(2.9-24.3)	12.9	(3.4-24.1)	−0.5	0.94
**Home**	3.7	((1.8-6.9 )	3.1	(1.8-5.1)	−1.0	0.17

Bold: significant difference p < 0.05.

^a^Differences were estimated using multilevel adjusted for differences in age, BMI, number of valid days & time in domain. School included to account for clustering.

^b^Differences were estimated using multilevel adjusted for differences in gender, BMI, number of valid days & time in domain. School included to account for clustering.

IQR, Inter Quartile Range; MVPA, Moderate to Vigorous Physical Activity, PE, Physical Education.

## Discussion

This study applied the SLOTH theoretical model [5] to examine GPS and accelerometer measured PA in the leisure, school, transport and home domain, and further expanded the model to encompass 11 subdomains [6]. MVPA was detected in all domains and subdomains, with almost 90% of all week day MVPA being objectively assigned to domains and subdomains. This is a significant improvement when compared with a recent study which used a similar methodology (only 60% of MVPA was assigned to locations or transport [10]).

There was large variation in how often subdomains were used for MVPA. In active transport, almost all participants accumulated some MVPA, while less than half of participants accumulated MVPA at playgrounds. There are a number of possible explanations for these findings; a) a large number of participants did not visit some subdomains; b) participants did not accumulate any MVPA whilst visiting some subdomains; or c) the employed data collection period was not suited to capture the use of certain subdomains (i.e., subdomains such as playgrounds that are used infrequently (i.e., less than weekly) but may be important places for accumulating MVPA). In support of our findings Qiugg and colleagues found that the overall amount of PA occurring at playgrounds was quite low [16]. However, in the current study, the proportion of time spent in MVPA when visiting a playground was relatively high (21-35%). Research focusing on the use of, and PA accumulated in subdomains such as playgrounds, sports facilities or urban green space, however, may need to employ a longer data collection period to capture behaviour in these subdomains.

In support of previous research we found that school and leisure are important domains in regard to the total amount of MVPA accumulated [10,12,15,34,35]. The proportion of time spent in MVPA when at a specific location may warrant attention as this can highlight domains or subdomains where PA promotion may be most efficient. The proportion of time spent in MVPA was found to be particularly high in active transport, playgrounds, sports facilities and urban green space. Other studies have found that active transport to school can contribute to children’s overall PA levels [36] and that the proportion of time spent in MVPA in active transport to school can be as high as 50% [19]. The current study found that active transport overall accounts for almost 20% of all MVPA and the average proportion of time spent in MVPA in active transport is between 30-35%. Rainham and colleagues [10] found similar results for their suburban population when investigating locations for youth PA. But contrary to our findings, girls achieved more MVPA than boys [10]. These differences may be due to GPS processing methods, or that the Rainham study assessed total amount of MVPA across valid days, without adjusting for differences in accumulated time or number of valid days between groups.

It is well established that in general, boys are more physically active than girls [6,15], however, gender differences in context specific PA behaviours has seldom been investigated. Our findings suggest that gender differences in PA may be partly explained by a generally lower level of PA among girls throughout the day and not correlated to specific behaviours, environments or contexts (home being the exception as no differences were observed). A recent study using accelerometer and GPS, found similar results to our study however the participant age range was younger (i.e., 6–11 year old children) [35]. Overall, these findings suggest that intrapersonal factors and social, cultural and perceived environments may be more influential in shaping girls PA behaviour than physical environment features of specific domains or subdomains.

Compared with adolescents, children accumulated more overall MVPA, and this was primarily explained by differences in MVPA accumulated during school, in particularly during recess whereas no age difference was detected during PE. In Denmark, children from grade 7 are permitted to leave the school grounds which may result in more sedentary activities (such as sitting in a café). Further, as children age they may prefer to socialize with peers as opposed to “playing”, which may also induce more sedentary behaviours. Thus, interventions aimed at reducing the decline in PA from childhood to adolescence within the school setting may be important; for example providing active programs for recess and keeping students on campus. Very few studies have examined correlates for adolescents PA during recess [37] and no intervention studies targeting adolescents were found in a recent review investigating the effect of school recess interventions on PA [38]. Future research in school settings should investigate adolescent school behaviour, in particular correlates for recess PA behaviour as well as effects of recess interventions. The lack of an age difference in MVPA during PE may be explained by PE being mandatory. Interventions aiming to increase the number of PE lessons as children become adolescents may thus help prevent or reduce the age related decline in PA. However, a recent prospective study concluded that the importance of the after school period for accumulating MVPA increases with age [39] and a strategy focusing on increasing PA during both the school and leisure domain may be warranted. Future prospective studies should also consider using a domain approach to track the relative and cumulative importance of domains and subdomains of MVPA from the early years through to adolescence.

Furthermore children’s MVPA patterns over the weekend were collected because different patterns of PA have been observed between week and weekend days [12]. In supplementary analyses of weekend data (not shown) it was found that all groups (boys, girls, children and adolescent) had fewer minutes of MVPA compared with week days (p < 0.05), that the gender pattern prevailed (boys compared to girls were significantly more active in all domains except the home domain, p < 0.05), but that no age differences were present (p > 0.1).

### Strengths and limitations

The current study pre-defined potential domains and subdomains relevant for children’s MVPA based on a theoretical model and published literature, and used a systematic approach to objectively identify and assess locations for PA. Considering the relatively novel application of accelerometer and GPS data to health research, the strength of this study was its large sample of detailed and matched accelerometer and GPS data. The use of accelerometers and GPS has limitations. The PALMS algorithms used in this study currently provides 80% accuracy in detecting travel modes. Also the GPS signal inaccuracy varies with the environment (e.g., urban canyons can reduce accuracy). As this study is conducted in an inner city environment, some locations and travel modes are likely to have been misclassified, and further studies are needed to determine the impact of different environments on GPS inaccuracy and further improve the PALMS algorithms. Accelerometer measurements also have limitations predominantly associated with data processing decisions and the choice of a threshold to determine the level of MVPA. As the aim of this study was to describe patterns between domains and subdomains by age and gender, the actual threshold value chosen is unlikely to impact the results. The subdomains included in this study were also derived to reflect places where children and adolescents accumulate MVPA. However, future research may also consider the inclusion of context specific domains for other activity intensities such as sedentary, light or vigorous activity, and the inclusion of counts per minute as an outcome variable.

The study sample was from a deprived area, which is rare in studies of this nature. The included schools are urban schools situated in one of the densest areas in Denmark, with a high proportion of ethnic minorities [22] and a high unemployment rate [21]. As such the results may differ for suburban and rural schools, or schools situated in more affluent areas. Future research should apply the PA behaviour measurement methods developed as part of this study to children living in a wide range of socio-economic areas and countries. This study did not aim to provide population estimates of domain specific PA among Danish children and adolescents as it was based on a sample from deprived area. As such the generalizability of this study is limited to children from schools in deprived neighbourhoods with a high unemployment rate and a high proportion of immigrants and descendants. However, the methodology developed as part of this study provides a significant advancement in the measurement of children’s PA behaviour which will advance the promotion of PA and inform new PA interventions. Focusing on a population in need, however, may have generated findings that would not have been seen elsewhere and helps demonstrate the utility of the current methods. This study included outcome measures for two variables, each consisting of four domains and 11 subdomains. Thus Type I error (i.e., false significance) may have been an issue due to multiple testing. Moreover, data was only collected during early fall and late spring, where daylight and overall weather conditions were quite similar, and therefore it is unknown if the results are valid during the winter months [14].

## Conclusions

This study demonstrates the usefulness and value of examining PA patterns using domain and context specific objective measures and provides a new methodology for understanding children’s PA patterns. Our results show significant gender differences, with girls being less active in almost all domains and subdomains. Our findings also indicate that declining PA from childhood to adolescence may be driven by a decrease in adolescents PA during school. This may provide support for those advocating for compulsory PE during school and support strategies aiming at increasing adolescent PA during recess, as this may positively impact the age related decline in PA. The findings need to be confirmed in longitudinal studies and with other populations. Future studies should also consider the appropriate length of data collection and weighing the need for detailed context specific behavior against increased participant burden. Objectively measured patterns of PA across domains/subdomains will in the future be useful in informing ‘active’ planning policy and can also be used to tailor PA interventions and inform policies for promoting child PA.

## Competing interests

The authors declare that they have no competing interests.

## Authors’ contributions

CDK conceived and coordinated the study, contributed to its design, acquisition of data, data cleaning, framing the database, statistical analyses, and drafted the manuscript. JS designed the database, contributed to the acquisition of data, and processed GPS, accelerometer and GIS data. JT conceived the study, and participated in its design. HC and JK contributed with significant input to the outline of the manuscript and AKE came with statistical input. All authors revised the manuscript critically, and read and approved the final manuscript.
